# A mobile-based randomized controlled trial on the feasibility and effectiveness of screening for major depressive disorder: study protocol

**DOI:** 10.1186/s40359-024-02230-6

**Published:** 2024-12-18

**Authors:** M. M. E. Zandbergen, E. E. L. Jansen, L. J. Jabbarian, H. J. de Koning, I. M. C. M. de Kok

**Affiliations:** 1 Department of Public Health, Erasmus MC, University Medical Center Rotterdam, Rotterdam, the Netherlands; 2 MiSi NeuroPsy, Rotterdam, the Netherlands

**Keywords:** Randomized controlled trial, Major depressive disorder, Screening Interventions, Public Mental Health, Patient Health Questionnaire-9

## Abstract

**Background:**

Mobile-based screening interventions to detect and treat Major Depressive Disorder (MDD) at an early stage might be a promising approach for reducing its societal burden. In the present study, we will evaluate the feasibility and effectiveness of screening for MDD using a mobile-based screening protocol.

**Methods:**

This study will be a three-arm, parallel randomized control trial (RCT) performed in a multi-ethnic population within the municipality of Rotterdam (the Netherlands). The trial includes two intervention groups that will be screened 4-weekly for MDD for 12 months using the Patient Health Questionnaire (PHQ-9) and a control group who does not receive mobile-based screening for MDD. Participants in the one-test intervention arm will be referred for further diagnosis and treatment, if necessary, after a single positive test score for moderate-severe major depression symptoms (PHQ-9 > 10). Participants in the multiple-test intervention arm will only be referred after three consecutive positive test scores. 1786 eligible participants will be included in the RCT, with 446 and 447 in the one-test and multiple-test referral arms, respectively, and 893 in the control arm. Primary outcome is participants’ QoL after 12 months (EQ-5D-5L). Secondary outcomes include participants’ QoL after 24 months (EQ-5D-5L), evaluating the occurrence and severity of MDD symptoms (PHQ-9), intervention engagement, and identifying public mental health differences based on sociodemographic characteristics, including age, gender, ethnicity, financial situation, educational background, and living area. Long-term results of the RCT will be incorporated into a microsimulation model to determine the long-term benefits, harms, and costs of MDD screening.

**Discussion:**

The information gained from examining the feasibility and (cost-) effectiveness of mobile-based screening for MDD could be of guidance for mental health policy implementations and support the introduction of mobile-based screening for MDD in the Netherlands and/or other nations.

**Trial registration:**

ClinicalTrials.gov: NL84280.078.23, NCT05989412, August 8, 2024.

**Supplementary Information:**

The online version contains supplementary material available at 10.1186/s40359-024-02230-6.

## Introduction

### Background

Major depressive disorder (MDD) is a common mental disorder, affecting more than 264 million people worldwide [1, 2]. MDD is the fourth-largest contributor to the global disease burden and the leading cause of disability worldwide [3]. The global prevalence of MDD is estimated to be 13%, which varies by age and gender, as well as socio-economic status [4]. Recurrences following an initial depressive episode are particularly problematic, with an estimated probability of 75 to 90%, because they often lead to a more severe course of illness, increased resistance to treatment, and greater overall impairment [5]. Consequences associated with MDD are widely spread: MDD is linked to a reduced (healthy) life expectancy and severely diminishes an individual’s Quality of Life (QoL), affecting their personal relationships, work life, and general health [1, 6, 7]. The total economic impact of MDD in the European Union is estimated to be more than 100 billion euros per year [8].

Given the long-term and recurrent nature of MDD and its numerous negative consequences, secondary prevention, alongside primary prevention, may be significantly important [9]. Secondary prevention of MDD relies on the early detection of individuals at risk, followed by timely and appropriate interventions to address symptoms before they develop into full depressive episodes. By intervening early, it is possible to alter the course of MDD, minimizing the chronic nature of the disorder and lowering the overall disease burden [10]. Screening can play a key role in timely identifying MDD, particularly in high-risk groups, who are unlikely to seek help or do not recognize symptoms of depression. A number of validated screening methods are available, and screening for depression is already recommended in several healthcare settings and population subgroups [11, 12]. In this study, we will use the Patient Health Questionnaire-9 (PHQ-9), a widely validated questionnaire for MDD screening, to detect depressive symptoms [11]. Previous studies suggest that screening may effectively reduce MDD symptoms and significantly increase remission rates [13].

The use of mobile-based screening tools presents an innovative approach to enhance MDD screening and improve mental health outcomes in managing MDD. Mobile-based interventions have the advantage of accessibility, scalability, convenience, and real-time data collection making them particularly suitable for populations that are less likely to engage with traditional healthcare [14–16]. While the potential for secondary prevention of MDD is expected [17–19], the effectiveness of mobile-based screening for MDD in the general adult and ethnically diverse population has not yet been shown. The present study aims to determine the feasibility and effectiveness of screening for MDD by providing a mobile-based screening protocol using a randomized controlled trial (RCT). The present trial will be the first experimental mobile-based screening intervention on MDD for the general, multi-ethnic adult population. We hypothesize our approach could provide new insights into early detection strategies and improve outcomes by reaching a broader and more diverse population, thereby offering scalable and flexible options for addressing the global burden of MDD.

### Objectives and hypothesis

This RCT assesses the feasibility and effectiveness of mobile-based screening for MDD in the adult population of Rotterdam, mainly the Southern part of the city. The primary aim is to compare changes in participants’ QoL between baseline and after 12 months in the screening versus those of the non-screening group. Secondary objectives involve measuring the occurrence and severity of MDD symptoms and quantifying the impact of screening in population subgroups. Additionally, the study aims to evaluate the long-term effects of MDD screening by measuring participants’ QoL after 24 months. These objectives will help to better understand the broader impact of mobile-based screening on mental health outcomes. A better understanding of the feasibility and effectiveness of mobile-based screening for MDD may be of guidance for mental health policymakers and may provide evidence for implementing MDD screening interventions.

We hypothesize that a mobile-based screening strategy for MDD will substantially reduce the burden of MDD over time, improve participants’ QoL, and minimize disparities associated with MDD.

## Methods/design

### Study design and setting

The MOOD (MObile-Based Screening fOr Depression) study is designed as a prospective, randomized controlled multi-arm trial with three parallel groups: a control group and two different intervention groups. We developed the MOOD intervention program by incorporating expertise from various stakeholders, including citizens and experts in mental, digital and public health. After completing a short survey to check for inclusion and exclusion criteria and the digital informed consent procedure, randomization will be performed at the individual level in a 2:1:1: ratio for the control group and the two intervention groups respectively. Participants in the control group will not receive mobile-based screening for MDD, while those in the two intervention groups receive a screening test using the PHQ-9 questionnaire at four-week intervals over a 12-month period. The intervention groups will be divided into two arms. In the one-test referral arm, participants who score positive for moderate to severe depressive symptoms (PHQ-9 score > 10) on a single screening will be referred for further diagnostic evaluation and treatment if necessary. In the multiple-test intervention arm, participants will only be referred for further diagnostic evaluation and treatment after three consecutive positive screenings indicating moderate to severe depressive symptoms (PHQ-9 score > 10). For the screening process in this study, we utilized a Clinical Trial Management System (CTMS) software developed by Your Research [20]. The application enhances efficiency by automating research processes such as data management, workflow optimization, and real-time reporting to ensure consistency and compliance throughout the study. Key features include error reduction, robust data encryption, comprehensive audit trails, ensuring data integrity and regulatory compliance. Participants can use this application at any moment to complete the survey(s). If any questionnaire is not finished after three days, participants will receive a reminder via email or push notification, depending on personal preferences. A second reminder will be sent after ten days. This allows participants to remain engaged and adhere to the study timeline. All study materials, including the website, questionnaires, Patient Information Form (PIF), and Informed Consent (IC) form, will be accessible in six languages: Dutch, English, Turkish, Spanish, Polish, and Arabic and will be provided in B1 language proficiency level. These languages were selected to ensure broad accessibility and inclusivity, reflecting the diverse linguistic backgrounds of the study population. Additionally, the questionnaires themselves will be available in 12 languages to further ensure the validity of the responses across the diverse population. The trial will be conducted by the Department of Public Health at the Erasmus University Medical Center Rotterdam, the Netherlands. The intervention area will focus on Rotterdam, particularly on the Southern part of the city, namely the districts Charlois, Feijenoord, and Ijsselmonde. The population in these districts is ethnically and socioeconomically diverse, with more than 90% having origins outside of Dutch heritage and more than 20% is unemployed [21]. It is expected that in this region there will be an above average prevalence of MDD, which will increase the screening’s potential impact and allow for better measurement [1, 6, 19]. This study is evaluated and approved by the Central Committee on Research Involving Human Subjects (CCMO) of the Netherlands (reference: # NL84280.078.23). The study is registered at the ClinicalTrials.gov Protocol Registration and Results System (ID = NCT05989412).

### Participants

We aim to include 1786 participants. Inclusion criteria are (1) be at least 18 years old; (2) live in the municipality of Rotterdam; (3) have access to a computer or smartphone; and (4) being able to complete the questionnaires in one of the provided languages. Exclusion criteria are (1) currently receiving therapy from a psychologist or psychiatrist for depressive symptoms; or (2) having been referred to a psychologist or psychiatrist in the past year for depressive symptoms. Eligibility screening will be conducted through the study’s website, after which participants will digitally sign the consent form via email. This will be followed by a baseline assessment completed through the application.

### Evaluation instruments

Evaluation instruments applied in the study are the PHQ-9 for measuring symptoms of depression and the 5-level EuroQol-5D (EQ-5D-5L) for assessing QoL, providing a comprehensive overview of the participants’ health status.

The *PHQ-9*, a widely used screening tool for detecting depression symptoms, is a validated and reduced version of the PHQ [11], which scores each of the 9 DSM-IV criteria as ‘0’ (not at all) to ‘3’ (nearly every day). As a severity measure, the overall score can range from 0 to 27, calculated by summing the scores for each question. Total scores of 5, 10, 15, and 20 represent the thresholds for mild, moderate, moderately severe and severe depression, respectively. MDD is suggested if ≥ 5 out of 9 items have been present at least ‘more than half the days’ in the past two weeks [11]. A PHQ-9 score ≥ 10 will be used in this study as referral advice threshold. This threshold has been shown to have a sensitivity of 88% and a specificity of 88% for MDD [11]. One of the 9 symptom criteria (“thoughts that you would be better off dead or of hurting yourself in some way”) counts as major depression if present at all, regardless of duration. In response to question 9, participants who select “more than half the days” will receive an additional notification including suicide prevention information. Given that the questionnaire depends on patient self-report, a healthcare professional should verify questionnaire outcomes and provide participants with diagnosis and further treatment if necessary. The PHQ-9 has been validated in the languages used in this study, including Dutch [22], English [11], Spanish [23], Polish [24], Turkish [25], and Arabic [26]). The English version of the PHQ-9 can be found in an additional file (see Additional file 2).

The *EQ-5D-5L* is being used as a 5-item self-report validated questionnaire for assessing health and health-related QoL across five core dimensions (5D): mobility, self-care, daily activities, pain/discomfort, and anxiety/depression and has been widely used to measure the QoL [27]. Items are scored on zero-five levels of severity (5L) for each dimension. Higher scores represent decreased QoL on that day [27]. The EQ-5D-5L can be found in an additional file (see Additional file 3). The EQ-5D-5L also includes the *EQ visual analogue scale* (EQ VAS), which captures participants’ self-rated health on a vertical visual analogue scale with endpoints labeled as’The best health you can imagine’ and ‘The worst health you can imagine’. The EQ VAS will be utilized as a quantitative measure of health outcome, reflecting the participants’ daily health-state perceptions [27]. The EQ VAS can be found in an additional file (see Additional file 3). The EQ-5D-5L has also been validated in the languages applied in this study, including Dutch [28]), English [29], Spanish [30], Polish [31], Turkish [32] and Arabic [33].

### Procedure

Participants will be recruited through different online and offline strategies to encourage citizens’ participation according to different cultural backgrounds. This approach ensures inclusivity and reflects the diverse population we aim to engage in the study. Offline recruitment includes in-person interactions with community members called “key figures”, video promotion, presentations and personally addressing people in public areas. Online recruitment efforts will include websites, social media networks, and local newspapers. By utilizing both digital and physical recruitment strategies, we aim to maximize reach and engagement. Interested individuals can enroll by initiating an application procedure through the study’s website [20], where they will complete an application form to assess eligibility. Eligible participants will then be invited to create a secure personal account and will be provided with a digital patient information form (PIF) and a digital informed consent (IC) form (see Additional file 1). The IC procedure is conducted digitally using Valid Sign, a digital signature platform that enables secure signing of documents online and streamlines the consent process. In line with our aim of ensuring simplicity and accessibility, an invitation email will provide instructions on downloading the Your Research CTMS application to their tablet or smartphone. This application will serve as the primary platform for all study related activities. Results will be sent directly to the server and will be accessible to participants at any time through the app. There will be a variety of platforms provided for additional inquiries about the study procedures including mail, WhatsApp or phone calls. Participants who complete and return the signed e-consent form will be enrolled and randomized in the trial. Depending on their assigned intervention arm, participants will be required to fill in questionnaires, sent via the Your Research app, within two weeks. To ensure adherence, push notifications will be sent to participants who do not complete the questionnaire within 3 days. Participants can choose their preferred notification method, which may include email, SMS, or application notifications. The data collection will run for 24 months. Participants, including those in the control group, will complete the EQ-5D-5L questionnaire via the Your Research CTMS application at baseline (0 months), and at 6, 12, and 24 months. Recruitment will be closely monitored to ensure that the target sample size is achieved. Once the sample size is reached, inclusion will be stopped. Participants will receive compensation at the end of the study based on the number of completed questionnaires, with a maximum of €50 (paid in gift cards). Additional vouchers may be earned by inviting more participants via the app. Participants can withdraw from the study at any time during the trial. To address any logistical or general MDD-related queries, participants can visit the study website [20] or contact the researchers by e-mail or WhatsApp. Additionally, a phone line will be available for inquiries on weekdays, staffed by Erasmus MC MOOD study researchers. To ensure participant safety, the program includes specific measures for addressing potential risks. For example, if questionnaire responses indicate severe depressive symptoms or a risk of self-harm, participants will be immediately directed to appropriate crisis support resources, such as ‘De Luisterlijn’ or ‘113 suicide prevention’ [34]. Additional information on suicide prevention and emergency contacts will be provided as needed, based on the assessment results. These safety measures are crucial for addressing any emerging mental health concerns in real-time, ensuring participant well-being throughout the trail.

**Fig. 1:**
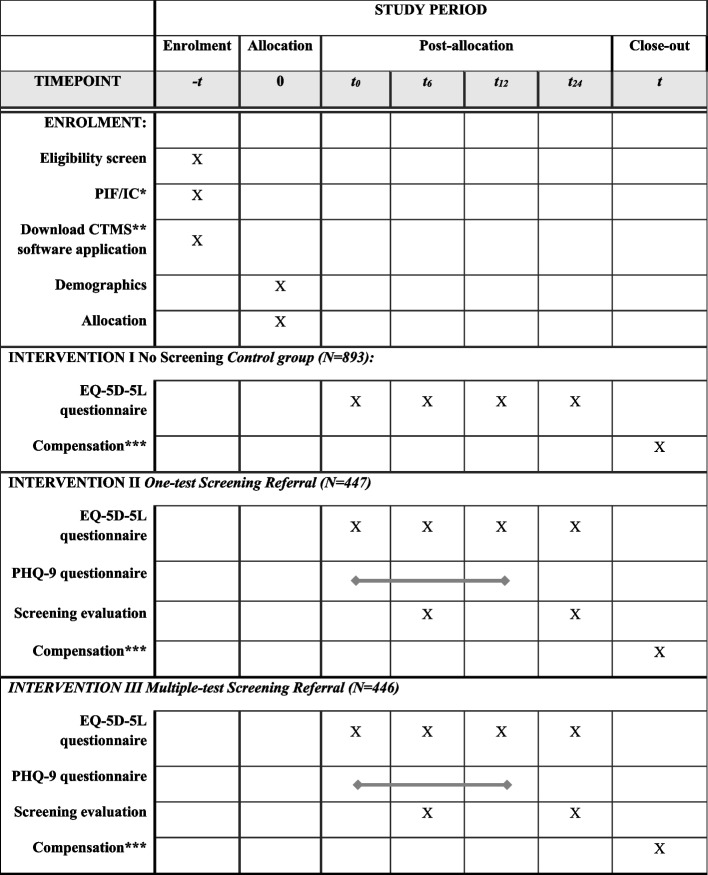
SPIRIT figure. A cross indicates one-time (screening) measurement. A bold line represents a 4-weekly screening measurement. *Patient information form/Informed consent. **Clinical Trial Management System *** Compensation preference will be assessed during the demographics assessment

The protocol and this manuscript were written following SPIRIT [35] guidelines. A detailed explanation of the screening timeline and intervention components is illustrated in SPIRIT Fig. 1.

### Randomization

Randomization will be performed by the Your Research CTMS software application as block randomization with a 2:1:1 allocation using stratification for age, gender, and living area. Due to the nature of the intervention neither participants nor staff can be blinded to allocation after randomization. A case report form for each participant will be prepared and labeled only with the participant number. A consent and identification list with numbers will be stored by Your Research. The two intervention groups will remain coded throughout the analysis phase.

### Outcomes measures

#### Primary outcome measures

The primary endpoint analysis (12 months) will be a comparison of outcomes in QoL for participants assigned to the control group and the combined intervention groups. The EQ-5D-5L total score differences between the baseline measurement (T0) and the post-intervention measurement at 12 months will be used to evaluate the outcome. This primary outcome measure allows us to broaden the impact of the intervention on participant’s’ overall well-being.

#### Secondary outcome measures

1. QoL: assessing participants’ QoL at baseline (T0) and post-intervention measurement (T24 months) using EQ-5D-5L total scores. Additionally, the last question of the EQ-5D-5L will be examined separately since it focuses on mental health. The EQ VAS will also be used to capture participants’ self-rated health, providing an additional measure of overall health perception.
2. Symptoms of depression: evaluating the occurrence and severity of MDD symptoms using the nine-item PHQ-9.
3. Screening adherence: the adherence to the screening intervention will be examined by assessing the proportion of participants completing the prescribed tests, aiming to understand the feasibility of the screening strategy.
4. Screening experiences: participants’ experience with the screening strategy will be evaluated through self-reported questionnaires after 6 and 24 months.
5. Population differences: the study will identify differences in MDD symptoms within the population based on social demographic characteristics, including age, gender, ethnicity, education level, living area and financial situation.

### Sample Size

In this study we compare the control group with both combined intervention groups with one-test referral for further diagnosis and treatment, and with multiple-test referral follow-up for further diagnosis and treatment. A total of 1,428 participants are required for the study, utilizing a 2:1:1 randomization scheme. This sample size will provide 80% power to detect a difference corresponding to an improvement of 0.40 in QoL. Additionally, frequent screening is expected to be effective in 40% of participants diagnosed with MDD, with a significance level set at 5% [30, 36–38]. Furthermore, a cumulative incidence of MDD during 12 months of 10% was assumed in our high-risk population [27, 39, 40]. These numbers lead to an expected difference in QoL of 0.016 at 12 months between the screening and control groups. A standard deviation of 0.18 in the score of the EQ-5D-5L in the general population has been expected [30, 41]. In the analysis, adjustments were made for the baseline EQ-5D-5L, assuming a correlation coefficient of 0.8 between the baseline measurement and the measurement at 12 months [42]. The design effect for adjusting for the EQ-5D at baseline is hence 0.36. As a result, calculating the sample size using the design effect for a two-sample t-test indicates that 714 participants are required in the control arm and combined intervention arms. To compensate for the potential 20% loss [43] of follow-up in both arms, we aim to recruit an additional 179 participants for the control arm and 179 participants for the intervention arm (1:1 between both intervention groups). To reach our goal we therefore need to include 1,786 participants in total.

### Data collection and management

Primary and secondary outcome measures for the MOOD trial will be collected using mobile-based questionnaires. Data will be consistently collected and stored securely using encrypted digital files in password-protected folders, accessible only to a limited number of researchers. The secure handling of participant data is central to maintaining confidentiality and integrity throughout the study. Participants will be assigned unique identification numbers for confidentiality. Contact information will be kept separate from other research data gathered throughout the study. Following the study’s completion, all data will be archived safely for 15 years at Erasmus Medical Center. Once all outcome data has been collected, it will be exported to a statistical program. All data will be kept and stored following the Personal Data Protection Act.

### Statistical analysis

At baseline, descriptive statistics will be utilized to describe participant characteristics, including demographic variables. This baseline assessment provides a foundation for understanding the distribution of key characteristics across the study groups. The statistical analysis for QoL at 12 months will use a standard linear regression model, correcting for baseline QoL measurements and other determinants. Multiple-testing correction will be applied, with the primary focus on identifying differences after 12 months between the control group and combined intervention groups. The secondary analysis utilizes a linear mixed model, incorporating interactions between time points and interventions to investigate screening effects over various time points. It assumes similar effects for both interventions, while interaction terms assess potential differences between intervention arms. This allows us to capture the nature of participant’s responses to the intervention over time. No missing values are anticipated for both the EQ-5D-5L and the PHQ-9 due to mandatory completion. However, there is a potential for entire questionnaire rounds to be missed, thereby resulting in missing data. To mitigate the impact of potential missing data, we will address it using mixed models. Included in the analysis are explanatory variables such as age, gender, ethnicity and other relevant factors readily available for consideration. Table 1 provides a summary of methods of analysis for each variable.

**Table 1:** Variables, measures and methods of analysis

**Variables/outcome**	**Hypothesis**	**Outcome Measures**	**Methods of Analysis**
**1) Primary**			
QoL at 12 months	Intervention improves QoL from baseline to 12 months	EQ-5D-5L total score differences from baseline to 12 months	Linear regression, correcting for baseline
**2) Secondary**			
QoL at 24 months	Intervention improves QoL from baseline to 24 months	EQ-5D-5L total score differences from baseline to 24 months, mental health question separately	Linear mixed model, interactions between time points and interventions
Depression symptoms		PHQ-9 scores	T-test or ANOVA
Intervention adherence		Proportion of participants completing tests.	Descriptive
Screening experiences	Improvement occurred	Self-reported questionnaire scores after 6 and 24 months	Descriptive statistics, T-test or ANOVA
**3) Subgroup analysis**			
Female vs Male	Sex affects outcomes		Regression methods with appropriate interaction terms
Age	Age affects outcomes		
Level of education	Low education affects outcomes		
Employment status	Employment status affects outcomes		
Living area	Living area affects outcomes		

Important remarks: in all analysis results will be expressed as coefficient, standard errors, corresponding 95% and associated *p*-values

## Discussion

The number of patients with MDD has been increasing over the last decades [1, 2], and therefore a significant number of people experience disability worldwide [3]. Our study is designed to address the growing need for effective screening methods to support early detection of MDD. While the general importance of MDD screening has been well established [17–19], there is a gap in knowledge regarding the optimal settings and approaches to maximize the impact of such interventions. Through our RCT, we aim to assess the feasibility and effectiveness of mobile-based screening for MDD, targeting a high-risk population. This study also provides an opportunity to examine how mobile-based health tools can bridge existing gaps in access to mental health services, especially in underserved or vulnerable communities.

A key strength of this study is its inclusive design, which ensures accessibility for participants from diverse linguistic and social-economic backgrounds. By developing study materials in six languages (English, Dutch, Spanish, Polish, Turkish, and Arabic), we aim to reduce language barriers and promote broad participation, ensuring that underrepresented groups have equal access to mental health services. Furthermore, all written content is presented at a B1 language proficiency level. This approach minimizes linguistic barriers and maximizes participant engagement across different ethnic and cultural groups, promoting inclusivity and enhancing the study’s generalizability. Another strength is the scalability of the intervention, which makes it feasible for wider implementation if proven effective. Additionally, the inclusion of a second intervention arm allows us to observe outcomes without direct referral, potentially improving the specificity of the intervention’s impact.

Transitioning from the broader context of screening, it is crucial to acknowledge specific challenges and strategies inherent to our study design. The first challenge that this study faces is the number of participants required. If we are unable to recruit the target population of 1,786 participants within the specified 12 months inclusion period, two alternatives will be considered: 1) Prolonging the inclusion time, and 2) expanding the recruitment area to encompass the entire municipality of Rotterdam.

Due to the power calculation and the high dropout experiences from earlier studies, it is necessary to include all 1,786 participants in the study. Dropout is a well-known risk in longitudinal RCTs [43], including studies focusing on major depression [44]. To avoid this risk, we will put effort into motivating the participants during the study. Therefore, we want to implement monthly newsletter updates in the application as well as updates about their personal gift cards.

Furthermore, it can be argued that administrating the PHQ-9 may act as a motivator for participants by providing them with valuable insight into their mental health status. This engagement may yield psychological benefits, fostering an improved sense of visibility, a feeling of being heard, and potentially reducing stigma surrounding depression—a positive influence on overall mental well-being. Research suggests that engaging individuals in self-assessment and reflection can lead to increased awareness and a proactive approach to mental health [45, 46]. Regular feedback provided through the app can reinforce participants’ active role in their mental health, potentially improving adherence and reducing dropout.

Another important consideration is the generalizability of our findings. While we focus on a high-risk population, the study’s results may have broader implications. We anticipate that our findings will help lay the foundation for future research into personalized screening for MDD. Our results could establish a groundwork for understanding risk factors associated with MDD, such as age, gender, ethnicity, or current financial status. Incorporating these factors into advanced prediction models could pave the way for developing a more personalized screening tool to predict the risk of MDD. This approach can enhance the identification and categorization of at-risk groups for MDD.

We hypothesize that mobile-based screening for MDD will lead to notable societal and clinical improvements. If this study can verify the feasibility and effectiveness of mobile-based screening for MDD in a high-risk population, it may provide evidence to support the implementation of screening for MDD in the general population. Given that our study focuses on a high-risk population, the next step would be to investigate whether similar results could be achieved in the general population, thereby potentially guiding mental health policymakers in their decision-making processes. This would also open opportunities to further explore the scalability of mobile-based interventions, assessing their long-term sustainability and their integration into existing health systems.

## Supplementary Information

Additional file 1.

Additional file 2.

Additional file 3.

## Data Availability

No datasets were generated or analysed during the current study.

## Abbreviations

DSM-IV: The Diagnostic and Statistical Manual of Mental Disorders, Fourth Edition, of the American Psychiatric Association
EQ-5D-5L: EuroQol-5D-5L, A standardized instrument for use as a measure of health outcome, functioning and health status
EQ-VAS: EuroQol Visual Analogue Scale, A standardized instrument for capturing self-rated health state
MDD: Major Depressive Disorder
PHQ9: Patient Health Quesionnaire-9, A standardized multipurpose instrument for screening the severity of depression
