# Supplementary material for: A mobile-based randomized controlled trial on the feasibility and effectiveness of screening for major depressive disorder: study protocol
Source: BMC Psychol. 2024 Dec 18;12:742. doi: 10.1186/s40359-024-02230-6 (PMC11657997; doi:10.1186/s40359-024-02230-6)
Supplement: Supplementary file 1 — Additional file 1. [file 40359_2024_2230_MOESM1_ESM.pdf]

# Test subject information for participation in medical scientific research

## Early detection of depression in Rotterdam-South through an app (MOOD Rotterdam).

*Mobile screening for major depressive disorder in adults from an ethnically and socioeconomically diverse population.*

## Introduction

Dear Sir/Madam,

This information letter is to ask you if you would like to participate in medical scientific research. Participation is voluntary. Here you can read about what kind of research it is, what it means to you, and what the advantages and disadvantages are. It is a lot of information. Please read the information and decide if you want to participate. If you decide to participate, please fill in the form in Appendix B.

### Ask your questions

You can use the information in this information letter to help you make your decision. We also encourage you to:
- Ask questions of the researcher giving you the information.
- Talk to your partner, family or friends about this research.
- Ask the independent expert questions. See Appendix A for contact details.
- Read the information at www.rijksoverheid.nl/mensenonderzoek.

## 1. General information

Erasmus MC set up this research. Below we will refer to Erasmus MC as the 'client'. Researchers, who may also be general practitioners/psychologists, carry out the research at Erasmus MC.

Participants in a medical research project are often called trial subjects. Both patients and healthy people can be subjects.

A total of 1786 subjects from Rotterdam were asked to participate in this research.
The research was approved by the medical ethics review committee.

## 2. What is the purpose of the research?

In this research, we are investigating whether we can use short questionnaires in an app to detect the early onset of depression. The advantage of this is that treatment can be started in time, which may prevent a severe depression.

## 3. What is the background to the research?

Depression is common and can have a significant impact on quality of life. About 20% of adults experience depression at some point in their lives. Several treatments are available, but they are currently underused. Screening could be a way to detect depression at an early stage and refer people for help. A questionnaire can be used to determine whether it is wise to seek help. In this research, we want to see if regularly administering questionnaires via an app is a good way to detect depression at an early stage.

## 4. How will the research go?

*How long will the research take?*
Will you take part in the research? If so, it will take about 2 years in total.

*Step 1: are you suitable to participate?*
The first thing we want to know is whether you are suitable to participate. So the app will ask you if you meet a number of criteria. The main ones are:

- You are 18 years or older
- You live in Rotterdam South
- You are able to give consent
- You are not currently under treatment by a psychologist or psychiatrist.
- You have not been referred to a psychologist or psychiatrist in the past year.

Please note: It may be that you are healthy but not suitable to participate. The researcher can tell you more about this by phone or email.

*Step 2: Use of the app*

For this research, we will make 3 groups:

- Group 1. People in this group are called every 4 weeks to complete a short questionnaire in the app. If they score high on the questionnaire or score positive on suicidal thoughts, they will be immediately referred for help. If consent was given in the consent form to inform the GP in case of referral, the GP will be informed by the researchers (446 participants).

- Group 2. People in this group will be called every 4 weeks to fill in a short questionnaire on the app. After three consecutive high scores on the questionnaire or a positive score on the suicidal thoughts, they will be referred for help. If consent was given in the consent form to inform the GP in case of referral, the GP will be informed by the researchers (447 participants).
- Group 3. People in this group will act as a control group and will not be screened for depression (893 participants).

A draw will determine which of the 3 groups you are assigned to.

The app will only be used as a screening method during this research. No diagnoses will be made through the app and therefore the app will not be used as a diagnostic tool.

*Step 3: studies and measurements*
We ask you to install the app and complete a questionnaire 4 times. You should complete the first questionnaire immediately. You will then be invited to complete the questionnaire again after 6, 12 and 24 months. The questions are about your quality of life. It will take you about 5 minutes to fill in the questionnaire.

If you are assigned to group 1 or group 2, you will also receive a mental health questionnaire every 4 weeks during the first year of the study. If you are referred for help, you will not receive this questionnaire. You will also receive a short questionnaire twice to evaluate the screening. These questionnaires will take about 5 minutes to complete each time.

*What is different from normal care?*
This research does not replace normal care. The aim of this research is to advise residents of Rotterdam-South to make use of this care at the earliest possible stage.

## 5. What arrangements do we make with you?

We would like the research to go well. Therefore, we will make the following arrangements with you:

- You will complete questionnaires via the app for the duration of the research.
- You will not take part in any other medical scientific research on your mental health during this research.
- You will complete the questionnaire each time the app asks you to do so.
- You must contact the researcher in these situations:
  - You are admitted to hospital
  - You suddenly experience health problems
  - You no longer wish to participate in the research.
  - Your phone number, address, or email address changes.

## 6. What side effects, adverse effects or discomforts may you experience?

*What are the possible inconveniences of measurements during the research?*

- You will be asked several times during the research to complete a short questionnaire in the app. This will take a few minutes of your time, as indicated in point 3.

## 7. What are the advantages and disadvantages of participating in the research?

Taking part in the research may have advantages and disadvantages. These are listed below. Think about them carefully and discuss them with others.

Screening may detect depression early, so that you can be referred for help early, but this is not certain.

Participating in the research may have these disadvantages or consequences
- Taking part in the research will take time.
- You will have to stick to the arrangements made in the research.
- The questionnaires may be confrontational.

*Don't want to participate?*
You make the decision to participate in the research. Don't want to take part? You can always seek help from your GP if you have mental health problems.

## 8. When will the research end?

The researcher will let you know if there is any new information about the research that is important to you. The researcher will then ask you if you want to continue to participate.

The research will stop for you in the following situations:
- All the studies according to the schedule are over.
- You want to stop the research yourself. You can do this at any time. You can do it yourself through the app. You do not have to say why you want to stop.
- The researcher thinks it is better for you to quit. The researcher will still invite you to a follow-up visit.
- One of the following bodies decides that the research should be stopped:
    - Erasmus MC,
    - the government, or
    - the medical ethics committee reviewing the research.

*What happens if you stop the research?*
The researchers use the data collected up to the point of stopping.

## 9. What happens after the research?

*Will you get the results of the research?*
Six (6) weeks after the research, the researcher can tell you which group you were in. Do you not want to know? Then tell the researcher. He/she will not tell you. About 12-18 months after the research is completed, the researcher will tell you the main results of the research.

## 10. What do we do with your data?

Do you want to take part in the research? Then you agree to the collection, use and storage of your data.

*What data do we keep?*

We store these data:
- your gender
- the first four digits of your postal code
- your age
- your e-mail address
- your mobile phone number
- your marital status
- your education level
- your income level
- your ethnic background
- details about your health
- data we collect during the research

*Why do we collect, use and store your data?*

We collect, use and store your data to answer the questions in this research. And to publish the results. The data may be used by the client to carry out the study and analyse the research data. Your mobile phone number will not be used by third parties and will only be used by the MOOD study to send you a reminder.

*How do we protect your privacy?*

To protect your privacy, we give your data a code. We will write only this code on all your data. We keep the key to this code in a secure place in the hospital. Whenever we process your data, we will only use this code. Even in reports and publications about the research, no one will be able to tell that it is about you.

*Who can see your data?*

Some people do have access to your name and other personal data without a code. These are people who check that the researchers are conducting the research properly and reliably. The following individuals can access your data:

- Members of the committee that monitors the safety of the research.
- An auditor working for the client.

These people will keep your data confidential. We will ask for your permission for these people to inspect your data. The Health and Youth Care Inspectorate may inspect your data without your consent.

*How long do we keep your data?*
We keep your data in the hospital for 15 years.

*Can we use your data for other research?*
The data collected may also be important for other scientific research in the field of mental health and/or for the development of the screening method. For this purpose, your data will be kept at the hospital for 15 years. You will indicate whether you agree to this on the consent form. Do you not agree? You can still take part in the research. You will receive the same care.

*What happens when unexpected discoveries are made?*
The researchers do not foresee any unexpected discoveries.

*Can you withdraw your consent to the use of your data?*
You can withdraw your consent to the use of your data at any time. Please tell the researcher. This applies to use in this research as well as use in other research. But note: if you withdraw your consent and researchers have already collected data for a research, then they can still use that data.

*Want to know more about your privacy?*

- Want to know more about your rights regarding the processing of personal data? Then have a look at www.autoriteitpersoonsgegevens.nl.
- Do you have any questions about your rights? Or do you have a complaint about the processing of your personal data? If so, please contact the person responsible for processing your personal data. For your research, this is:
    - Erasmus MC. See Appendix A for contact details, and website.
- If you have a complaint about the processing of your personal data, we recommend that you first discuss it with the research team. You can also contact the data protection officer of Erasmus MC. Or you can lodge a complaint with the Dutch Data Protection Authority.

*Where can you find more information about the research?*
The following website(s) provide more information about the research. www.yourresearch.nl and/or www.moodstudie.nl. Following the research, the website may display a summary of the results of the research. You can find the research by searching www.moodstudie.nl.

## 11. Will you receive compensation if you participate in the research

Using the app for the research will not cost you anything. For participating in this research, you will receive a voucher of your choice at the end of the study. You can choose from the following options: Bol.com, Albert Heijn, VVV, cadeakaart.nl. If you complete all 4 quality of life questionnaires, you will receive €15. Groups 1 and 2 also receive €2.50 for each completed screening questionnaire. You can also increase your voucher by inviting other participants to the research. This can be done through the app.

## 12. Are you insured during the research?

You do not have additional insurance for this research. This is because there are no additional risks involved in taking part in this research. Therefore, Erasmus MC is not required by the medical ethics committee to take out additional insurance.

## 13. Informing your GP

The researcher will not contact your GP during the research. If during the research it becomes apparent that it would be useful to contact your GP, you will be advised to do so yourself.

## 14. Do you have any questions?

You can ask the research team questions about the research. You can do this by calling 010-7045699 between 10am and 5pm Monday to Friday. You can also send an email to mood@erasmusmc.nl.

Do you want advice from someone who has no vested interest? Then contact the Independent Expert (see Appendix A for contact details). This person knows a lot about the research but is not involved in it.

Do you have a complaint? If so, discuss it with the researcher or doctor treating you. Would you rather not do this? Then go to the Erasmus MC Complaints Committee. Appendix A shows where you can find it.

## 15. How do you consent to the research?

You can take some time to think about your participation in this research. You will also be able to ask the researcher questions if you do not understand the information you have been given or if you would like to know more. Do you want to participate? Then fill out the consent form included with this information letter. You and the researcher will both receive a signed version of this consent form by email.

Thank you for your time.

## 16. Appendices to this information

A. Contact details
B. Consent form(s)

## Appendix A: contact details for Erasmus MC

Lead researcher:
Dr. Inge de Kok
010-703 8460mood@erasmusmc.nl

Independent expert:

Dr. Eveline Heijnsdijk
010-703 8460
e.heijnsdijk@erasmusmc.nl

Complaints:
If you are not satisfied with the research or treatment, you can contact Erasmus MC's independent complaints department.
A digital complaint form is available on the Erasmus MC website at https://www.erasmusmc.nl/nl-nl/patientenzorg/klachtenopvang-en-klachtenbemiddeling.
After filling in the form, it will automatically be sent to the complaints officer.
If you are unable to complete the digital complaint form, you can also send your complaint by post: Erasmus MC, Secretariat for Complaints (GK-745), Antwoordnummer 55, 3000 WB Rotterdam.
Please include your name, patient number (if applicable), the name of the research and your contact details in the letter. On receipt of the letter, the complaints officer will contact you.

Data Protection Officer of the institution:
The Data Protection Officer of Erasmus MC can be contacted via the Secretariat of the Legal Affairs Department. E-mail: functionaris.gegevensbescherming@erasmusmc.nl Tel: 010-703 4986

For more information about your rights:
For more information or if you have any questions about your rights, please contact the Data Protection Officer or the Personal Data Authority.

## Appendix B: Subject Consent Form

Pertaining to

Early detection of depression in Rotterdam-South through an app (MOOD Rotterdam)

- I read the information letter. I was also able to ask questions. My questions were answered well enough. I had enough time to decide if I wanted to participate.
- I know that my participation is voluntary. I also know that I can decide at any time not to take part in the research. Or to stop. I do not have to say why I want to stop.
- I give the researcher permission to inform my GP if I score high on the questionnaire if I am assigned to Group 1 or Group 2.
- I give the researchers permission to collect and use my data. The researchers will only do this to answer the research question of this research.
- I know that some people will be able to see all my data for the purpose of monitoring the research. These people are listed in this information letter. I give these people permission to see my data for this research.
- I understand that my encrypted data will be sent to countries outside the EU where EU data protection laws do not apply. I consent to this.
- Please tick yes or no in the table below.

| | | |
|---|---|---|
| I consent to the retention of my data for use in other research as described in the information letter. | Yes ☐ | No☐ |
| I consent to being asked if I would like to take part in a follow-up research research after this research if necessary. | Yes ☐ | No☐ |
| I give the researchers permission to let me know which group I was in after the research. | Yes ☐ | No☐ |

- I want to participate in this research.

My name is (test subject): ………………………………..

Signature: ………………………  Date : __ / __ / __

------------------------------------------------------------------------------------------------------------

I declare that I have fully informed this test subject about the above research.

Will information become known during the research that could influence the test subject's consent? If so, I will inform the test subject in good time.

Name of researcher (or their representative): Inge de Kok

Signature:………………………  Date: __ / __ / __

------------------------------------------------------------------------------------------------------------

<if applicable>
Additional information was provided by:
Name:………………………………..
Function:………………………………
Signature:………………………            Date: __ / __ / __

-----------------------------------------------------------------------------------------------------------------

*The test subject will be given a full information letter along with a signed version of the consent form*
