# Supplementary material for: A mobile-based randomized controlled trial on the feasibility and effectiveness of screening for major depressive disorder: study protocol
Source: BMC Psychol. 2024 Dec 18;12:742. doi: 10.1186/s40359-024-02230-6 (PMC11657997; doi:10.1186/s40359-024-02230-6)
Supplement: Supplementary file 2 — Additional file 2. [file 40359_2024_2230_MOESM2_ESM.pdf]

PATIENT HEALTH QUESTIONNAIRE-9 (PHQ-9)

**Over the past 2 weeks, how often have you been bothered by any of the following problems?** *(Use "✔" to indicate your answer)*	**Not at all**	**Several days**	**More than half the days**	**Nearly every day**
**1.** Having little interest or pleasure in doing things	0	1	2	3
**2.** Feeling down, depressed, or hopeless	0	1	2	3
**3.** Having trouble falling or staying asleep, or sleeping too much	0	1	2	3
**4.** Feeling tired or having little energy	0	1	2	3
**5.** Having a poor appetite or overeating	0	1	2	3
**6.** Feeling bad about yourself — or that you are a failure or have let yourself or your family down	0	1	2	3
**7.** Having trouble concentrating on things, such as reading the newspaper or watching television	0	1	2	3
**8.** Moving or speaking so slowly that other people could have noticed? Or the opposite — being so fidgety or restless that you have been moving around a lot more than usual	0	1	2	3
**9.** Having thoughts that you would be better off dead or of hurting yourself in some way	0	1	2	3

FOR OFFICE CODING ___0___ + ______ + ______ + ______

=Total Score: ______

If you circled any problems, how difficult have these problems made it for you to do your work, take care of things at home, or get along with other people?

**Not difficult at all**	**Somewhat difficult**	**Very difficult**	**Extremely difficult**
☐	☐	☐	☐

Developed by Drs. Robert L. Spitzer, Janet B.W. Williams, Kurt Kroenke and colleagues, with an educational grant from Pfizer Inc. No permission required to reproduce, translate, display or distribute.
